# Prognostic impact of the number of metastatic lymph nodes after surgery in locally advanced hypopharyngeal cancer

**DOI:** 10.1186/s12885-022-10172-8

**Published:** 2022-10-27

**Authors:** Ari Nishimura, Tomoya Yokota, Satoshi Hamauchi, Yusuke Onozawa, Akifumi Notsu, Fuyuki Sato, Takeshi Kawakami, Hirofumi Ogawa, Tsuyoshi Onoe, Takashi Mukaigawa

**Affiliations:** 1 Division of Gastrointestinal Oncology, Shizuoka Cancer Center, 1007, Shizuoka, Japan; 2 Division of Medical Oncology, Shizuoka Cancer Center, 1007, Shizuoka, Japan; 3 Department of Biostatistics, Clinical Research Center, Shizuoka Cancer Center, 1007, Shizuoka, Japan; 4 Division of Pathology, Shizuoka Cancer Center, 1007, Shizuoka, Japan; 5 Radiation and Proton Therapy Center, Shizuoka Cancer Center, 1007, Shizuoka, Japan; 6 Division of Head and Neck Surgery, Shizuoka Cancer Center, 1007, Shizuoka, Japan

**Keywords:** Hypopharyngeal squamous cell carcinoma, Postoperative risk factor, Metastatic lymph nodes, Chemoradiotherapy, Extra-nodal extension

## Abstract

**Background:**

Postoperative chemoradiotherapy (CRT) is a standard therapy for patients with high-risk factors for head and neck squamous cell carcinoma, including positive margin and extra-nodal extension (ENE). However, the prognostic impact of the number of pathological metastatic lymph nodes (pLNs) in hypopharyngeal carcinoma (HPC) is unclear. Thus, this study aimed to investigate postoperative prognostic factors for locally advanced hypopharyngeal squamous cell carcinoma (LA-HPSCC) with a focus on the number of pLNs.

**Methods:**

We retrospectively analyzed medical records of 99 consecutive patients with LA-HPSCC who underwent total pharyngo-laryngo-esophagectomy (TPLE) and bilateral neck dissection (ND) between December 2002 and May 2019.

**Results:**

The median follow-up time for all censored patients was 63.2 months. The median overall survival (OS) was 101.0 months (95% confidence interval [CI] 48.1–134.9). patients had pLNs ≥ 3. Forty-six (45.5%) patients were diagnosed with ENE. Twenty (20.2%) patients received postoperative CRT. The multivariate analysis revealed that pLNs ≥ 3 (median OS: 163.2 vs. 31.8 months, hazard ratio [HR] 2.39, 95% CI 1.16–4.94, p < 0.01) and ENE (median OS: 161.0 vs. 26.3 months, HR 4.60, 95% CI 2.26–9.36, p < 0.01) were significantly associated with poor prognosis and that postoperative CRT (HR 0.34, 95% CI 0.16–0.72, p < 0.01) was significantly associated with better prognosis. The cumulative incidence of distant metastasis was higher in patients with pLNs ≥ 3 than in those with pLNs < 3 (p < 0.01).

**Conclusion:**

pLNs ≥ 3 and ENE were significant poor prognostic factors for patients with LA-HPSCC who underwent TPLE and bilateral ND.

## Background

Locally advanced hypopharyngeal squamous cell carcinoma (LA-HPSCC) is a malignant neoplasm with poor prognosis among all head and neck cancers, and patients with LA-HPSCC have a 5-year survival rate of 30–40% [1]. The standard treatment for LA-HPSCC is primary surgical resection, including total laryngo-pharyngo-esophagectomy (TPLE) with neck dissection (ND) or definitive chemoradiotherapy (CRT). Although CRT may preserve laryngeal function, local control and survival are poor in patients with a nonfunctional larynx or tumor penetration through cartilages into surrounding soft tissues [2]. Because LA-HPSCC often accompanies such extensive tumor invasion, many patients require ablative surgical resection.

However, because surgical treatment alone is often associated with high risks for locoregional recurrence and distant metastases, postoperative treatments are necessary for patients with high-risk factors [3]. The Radiation Therapy Oncology Group (RTOG) 95 − 01 and European Organization for Research and Treatment of Cancer (EORTC) 22,931 trials identified extranodal excision (ENE), positive margin, and multiple pathological metastatic lymph nodes (pLNs) as high-risk factors [4, 5].

In contrast, the combined analysis of RTOG 95 − 01 and EORTC 22,931 trials did not reveal that the number of pLNs was a high-risk factor for recurrence [6], although the proportion of patients with LA-HPSCC was small. However, Zumsteg et al. reported that the number of pLNs was a dominant determinant of survival of patients with non-oropharyngeal HNSCC [7]. Ho et al. demonstrated that increasing quantitative lymph node (LN) burden was associated with poor survival of patients with hypopharyngeal and laryngeal cancer [8]. Thus, the prognostic impact of the number of pLNs in patients with LA-HPSCC has been unclear. This study aimed to investigate postoperative prognostic factors for LA-HPSCC with a focus on the number of pLNs.

## Methods

### Study design and patients

Consecutive patients with clinical stage III or IV (American Joint Committee on Cancer TNM System, 8th edition) HPSCC who underwent TPLE as primary resection and bilateral ND at Shizuoka Cancer Center Hospital between December 2002 and May 2019 were selected. Patients who underwent prior radiotherapy (RT) for head and neck lesions or ND, those who received induction chemotherapy, those with Eastern Cooperative Oncology Group performance status (ECOG PS) of ≥ 2, and those with synchronous or metachronous malignancies within 5 years except for carcinoma in situ or intramucosal tumors were excluded. This study was approved by the institutional review committee of Shizuoka Cancer Center (Shizuoka, Japan) (J2021-79-2021-1-3) and complied with the standards of the Declaration of Helsinki. Written informed consent was obtained from all patients after the details of the study and its opt-out policy were explained.

### Adjuvant therapy

The decision regarding adjuvant therapy was made by a multidisciplinary tumor board and was based on the presence of ENE, ICR, number of pLNs, ECOG PS, primary site, and metabolic criteria.

### Evaluation

All clinical and follow-up data were obtained from electronic medical records. Pathological diagnosis was evaluated according to the American Joint Committee on Cancer TNM System, 8th edition. Pretreatment evaluations included medical history, physical examinations, laboratory tests, endoscopy, computed tomography (CT), magnetic resonance imaging (MRI), and [18 F]-fluorodeoxyglucose positron-emission tomography/CT fusion imaging. Disease was assessed using CT or MRI at 6–8 weeks after completing RT or when clinical signs suggested progressive disease.

### Statistical analysis

Overall survival (OS) was calculated from the date of surgical treatment to the date of last follow-up or death owing to any cause. Distant metastasis-free survival (DMFS) was calculated from the date of surgical treatment to the date of confirmed distant metastasis. OS and DMFS were calculated using the Kaplan-Meier method. Cox regression was used for univariate and multivariate analyses. Cumulative incidence curves were compared using the log-rank test, and two-sided p-values ≤ 0.05 were considered statistically significant. Statistical analyses were performed using EZR version 1.54 (Saitama Medical Center, Jichi Medical University, Saitama, Japan), which is a graphical user interface for R (The R Foundation for Statistical Computing, Vienna, Austria) [9].

## Results

### Patient characteristics

**Fig. 1:**
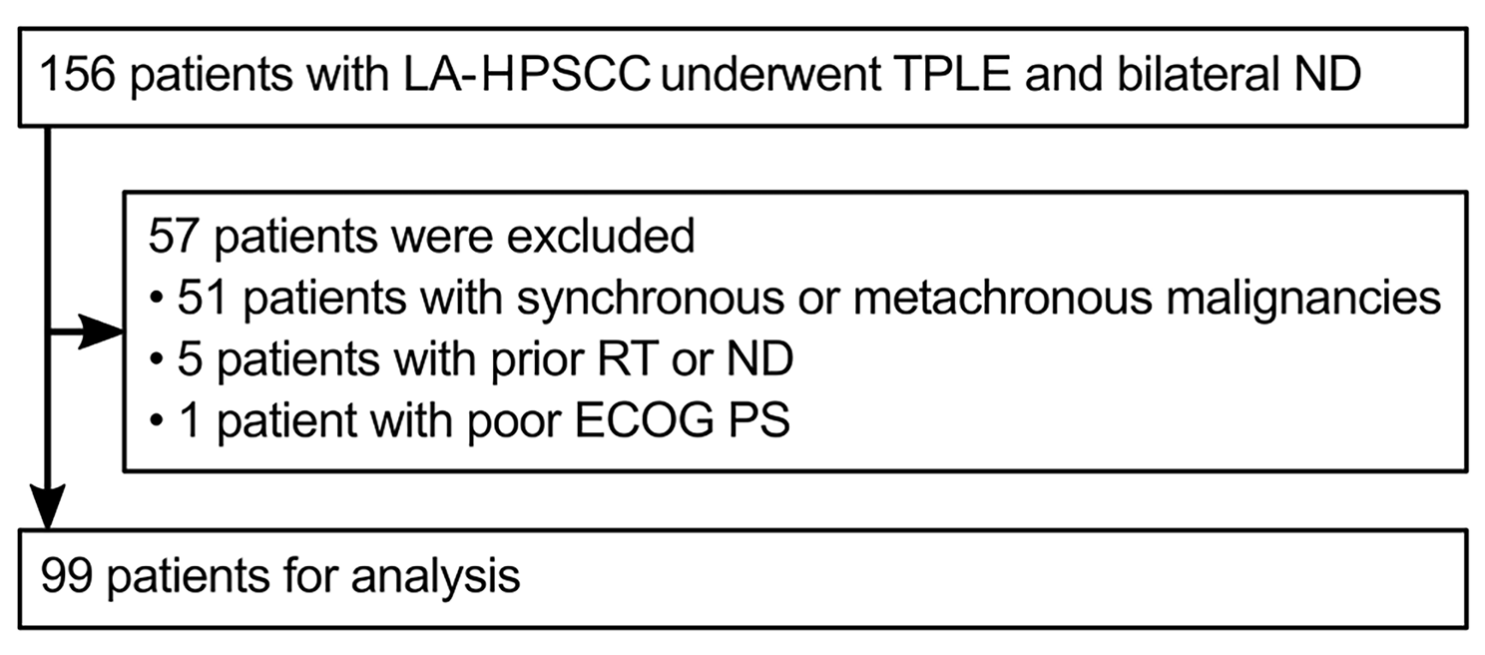
Patient selection criteria. A flow chart illustrating the composition of the study cohort (n = 99). *LA-HPSCC*: locally advanced hypopharyngeal squamous cell carcinoma, *TPLE*: total pharyngo-laryngo-esophagectomy, *ND*: neck dissection, *RT*: radiotherapy. *ECOG PS*: Eastern Cooperative Oncology Group performance status

Overall, 156 consecutive patients underwent and bilateral ND for LA-HPSCC at the Shizuoka Cancer Center between December 2002 and May 2019. Among them, 51 patients with synchronous or metachronous malignancies, 5 with prior RT or ND, and 1 with ECOG PS of 2 were excluded. A total of 99 patients were considered eligible and included in this study. Figure 1 shows a flow chart of patient selection.

Patient characteristics are shown in Table 1. The median age of the patients was 69 years (range: 41–89 years). All patients underwent TPLE with synchronous bilateral ND. The average number of total lymph nodes harvested was 74.6 (range: 28–178). There were 4 patients with carcinoma in situ or intramucosal tumors, including 1 breast cancer, 2 esophageal cancer, and 1 gastric cancer. The pathological diagnoses were ENE and ICR in 54 (53.5%) and 11 (11.1%) patients, respectively. Among 99 patients, 20 (20.2%), 31 (31.3%), 3 (3.0%), and 45 (45.5%) patients received adjuvant CRT, RT alone, adjuvant chemotherapy with S-1 (an oral fluoropyrimidine), and no adjuvant therapy, respectively. The number of pLNs was 0, 1, 2, 3, 4, and 5 in 15 (15.2%), 15 (15.2%), 15 (15.2%), 14 (14.1%), 9 (9.1%), and 31 (31.3%) patients, respectively.

**Table 1:** Patient characteristics

Characteristics		Patients (***n*** = 99)
Sex, n (%)	Male	87 (12.1)
	Female	12 (87.9)
Age, n (%)	≥ 65 years	69 (69.7)
	< 65 years	30 (30.3)
ECOG PS, n (%)	0	22 (22.2)
	1	77 (77.8)
Primary subsite, n (%)	Pyriform sinus	63 (63.6)
	Posterior wall	17 (17.2)
	Postcricoid region	19 (19.2)
pT stage, n (%)	T1–2	12 (12.1)
	T3–4	87 (87.9)
pN stage, n (%)	N0–2a	27 (27.3)
	N2b–2c	19 (19.2)
	N3b	53 (53.5)
Smoking history (pack-years), n (%)	≧ 10	77 (77.8)
	< 10	22 (22.2)
Habitual alcohol consumption, n (%)	No	16 (16.2)
	Yes	83 (83.8)
positive margin, n (%)	No	88 (88.9)
	Yes	11 (11.1)
ENE, n (%)	No	46 (46.5)
	Yes	53 (53.5)
Adjuvant therapy, n (%)	None	45 (45.5)
	RT alone	31 (31.3)
	Cisplatin + RT	13 (13.1)
	Carboplatin + RT	5 (5.1)
	FP + RT	2 (2.0)
	S-1	3 (3.0)

*ECOG*: Eastern Cooperative Oncology Group, *PS*: performance status, *ENE*: extranodal-extension, *RT*: radiotherapy, *FP*: 5-fuluorouracil + cisplatin

### OS and prognostic factors for OS

The median follow-up time for all censored patients was 63.2 months (range 5.8–196.9 months). The median OS for all patients was 101.0 months (95% confidence interval [CI]: 48.1–134.9). The 5-year OS, disease free survival and disease specific survival rate were 55.9% (95%CI: 44.9–65.6%), 51.0% (95%CI: 40.5–60.7%), and 62.2% (51.0-71.6%), respectively. Various cut-off values for the number of pLNs were tested using the log-rank test in the OS analysis. The number of pLNs of 3 could differentiate the shorter OS group (≥ 3) from the longer OS group (< 3) using the log-rank test (p < 0.01).

**Fig. 2:**
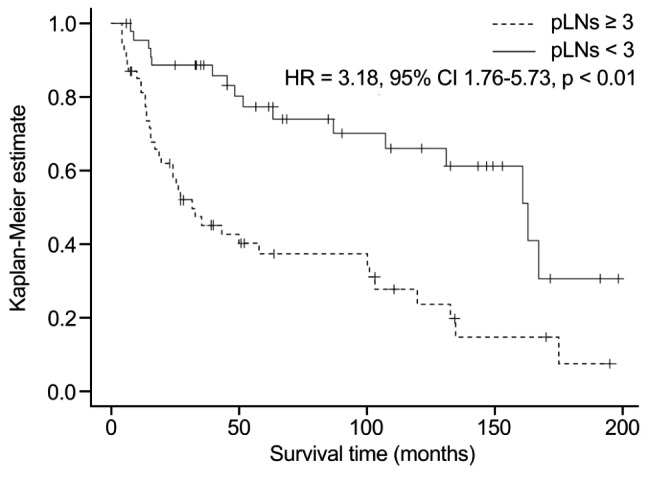
A Kaplan-Meier plot showing OS of patients with pathological metastatic regional lymph nodes ≥ 3. *HR*: hazard ration, *CI*: confidence interval

The univariate analysis revealed that age (≥ 65 years) (hazard ratio [HR]: 1.89, 95% CI: 1.01–3.52, p = 0.046), pLNs ≥ 3 (HR: 3.18, 95% CI: 1.76–5.73, p < 0.01), and positive ENE (HR: 3.66, 95% CI: 2.01–6.67, p < 0.01) were associated poor OS (Table 2). The multivariate analysis revealed that pLNs ≥ 3 (HR: 2.39, 95% CI: 1.16–4.94, p = 0.019) and positive ENE (HR: 4.60, 95% CI: 2.26–9.36, p < 0.01) were significantly associated with poor OS. A Kaplan-Meier curve for OS according to pLNs ≥ 3 is shown in Fig. 2. Adjuvant CRT (HR: 0.34, 95% CI: 0.16–0.72, p < 0.01) was associated with better overall prognosis.

**Table 2:** Factors associated with OS in the univariate and multivariate analyses

Factors	Category	Univariate analysis			Multivariate analysis		
		HR	95% CI	p value	HR	95% CI	p value
Sex	Male vs. female	1.30	0.55–3.05	0.550	1.18	0.49–2.86	0.711
Age	≥ 65 years vs. <65 years	1.89	1.01–3.52	0.046	1.95	0.98–3.65	0.056
pT	pT1–2 vs. pT3–4	1.95	0.83–4.60	0.126	2.11	0.86–5.18	0.103
Number of pLNs	≥ 3 vs. 0–2	3.18	1.76–5.73	< 0.001	2.39	1.16–4.94	0.019
Adjuvant therapy	CRT vs. any	1.13	0.59–2.17	0.714	0.34	0.16–0.72	0.005
Positive ENE	Yes vs. no	3.66	2.01–6.67	< 0.001	4.60	2.26–9.36	< 0.001
Positive margin	Yes vs. no	1.18	0.50–2.76	0.071	0.55	0.22–1.36	0.193

* h*: hazard ratio, *CI*: confidence interval, *pLNs*: pathological metastatic lymph nodes, *CRT*: chemoradiotherapy, *ENE*: extranodal-extension

### Comparison of outcomes between pLNs ≥ 3 and pLNs < 3

Among 99 patients, 42 (42.1%) had recurrence or metastasis. To investigate the reason why OS of patients with pLNs ≥ 3 was poorer than those with pLNs < 3, we then compared outcomes between pLNs ≥ 3 and pLNs < 3 by focusing on recurrent or metastatic sites in Table 3. Recurrent regional LNs were documented in 12 and 3 patients with pLNs ≥ 3 and pLNs < 3, respectively (22.2% vs. 6.7%, p = 0.047). Distant metastasis was documented in 20 and 6 patients with pLNs ≥ 3 and pLNs < 3, respectively (37.0% vs. 13.3%, p = 0.011). Among distant metastatic sites, patients with pLNs ≥ 3 had more lung metastasis than those with pLNs < 3 (33.3% vs. 11.1%, p < 0.01).

**Fig. 3:**
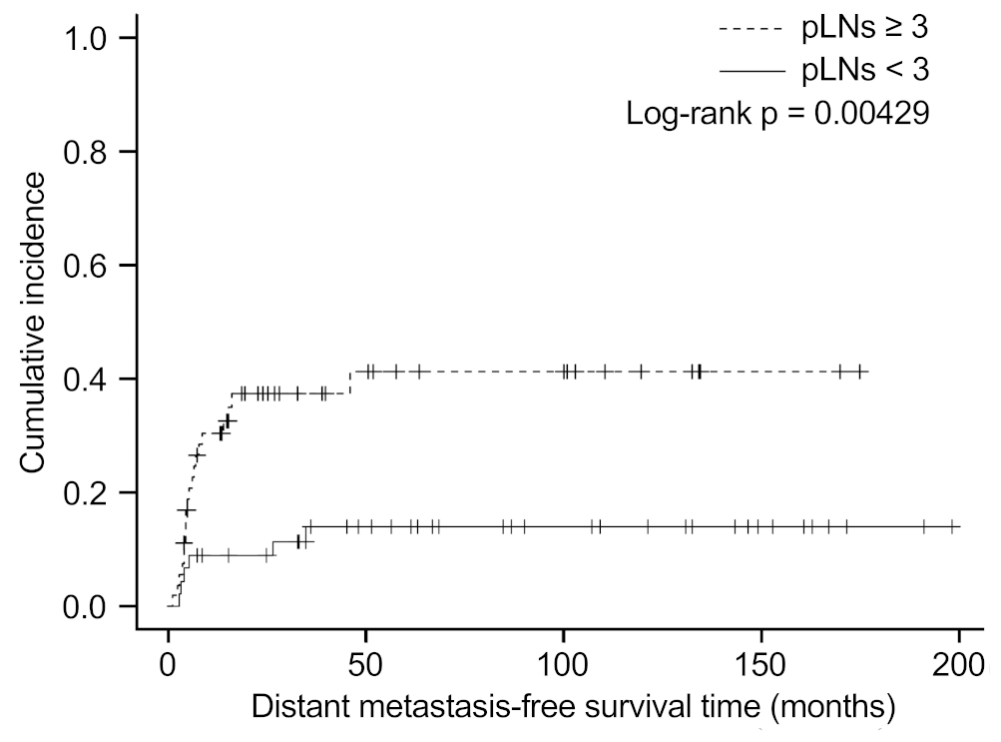
Cumulative incidence of distant metastasis for patients with pLNs ≥ 3 vs. those with pLNs < 3 (n = 26). Cumulative incidence of distant metastasis for patients with pLNs ≥ 3 was higher than that for patients with pLNs < 3 (log-rank p < 0.01). *pLNs*: pathological metastatic lymph nodes

The cumulative 1- and 3-year incidence rates of distant metastasis in patients with pLNs ≥ 3 were higher than those in patients with pLNs < 3 (30.4% vs. 11.4% and 37.3% vs. 14.0%, respectively; p < 0.01) (Fig. 3).

**Table 3:** Comparison of recurrent or metastatic sites between pLNs ≥ 3 and pLNs < 3

Site	All (n = 99)	pLNs ≥ 3 (n = 54)	pLNs < 3 (n = 45)	p value*
None	57 (57.9%)	21 (38.9%)	36 (80.0%)	0.01 >
Local	1 (1.0%)	1 (1.9%)	0 (0.0%)	1.00
Regional LNs	15 (15.4%)	12 (22.2%)	3 (6.7%)	0.047
Distant	26 (26.3%)	20 (37.0)	6 (13.3%)	0.011
Lung	23 (23.2%)	18 (33.3%)	5 (11.1%)	0.01 >
Bone	6 (6.1%)	5 (9.3%)	1 (2.2%)	0.213
Pleura	1 (1.9%)	1 (1.9%)	0 (0.0%)	1.00
Liver	1 (1.9%)	1 (1.9%)	0 (0.0%)	1.00
LNs	1 (1.9%)	1 (1.9%)	0 (0.0%)	1.00

*All p-values were obtained using Fisher’s exact test for pLNs ≥ 3 and pLNs < 3.

*pLNs*: pathological metastatic lymph nodes, *LNs*: lymph nodes

## Discussion

We analyzed clinical outcomes of patients with clinical stage III or IV LA-HPSCC who underwent primary surgical treatment and demonstrated that pLNs ≥ 3 and ENE were independent postoperative risk factors for patients for LA-HPSCC, while adjuvant CRT was associated with better survival.

Our results are in line with those of previous studies that demonstrated the ratio of metastatic to examined LNs and multiple pLNs are postoperative high-risk factors for LA-HPSCC [10–13]. Based on these studies, multiple pLNs should be considered a high-risk factor for postoperative recurrence or metastasis. Our study revealed that the cumulative incidence of distant metastasis was significantly higher in patients with pLNs ≥ 3 than in those with pLNs < 3. This result is consistent with findings of other reports that revealed that patients with LA-HPSCC having more advanced N classification had significantly worse distant control [14, 15]. Therefore, the high frequency of distant failure could contribute to worse prognosis in patients with LA-HPSCC having pLNs ≥ 3.

How should we approach LA-HPSCC patients with multiple lymph nodes metastasis to improve prognosis? The first perioperative treatment option is postoperative CRT. However, the combined analysis of EORTC 22,931 and RTOG 95 − 01 trials did not indicate an advantage of postoperative CRT over RT in patients with multiple LN metastases without ENE [6]. Therefore, postoperative CRT is not generally recommended for this population. Our study also demonstrated that postoperative CRT did not improve OS in patients with pLNs ≥ 3 (data not shown), although adjuvant CRT was associated with better overall prognosis in the overall population.

The second approach is induction chemotherapy (ICT) could be used as a more intensive perioperative treatment to control distant metastasis because the poor prognosis of patients with LA-HPSCC combined with the presence of a high number of pLNs may be explained by the high incidence of distant metastases. Several clinical trials have demonstrated that ICT with docetaxel, cisplatin, and fluorouracil improved DMFS in the setting of non-surgical treatment of locally advanced head and neck squamous cell carcinoma despite no improvement in OS [16–18]. Although the potential benefit of ICT remains unclear in the setting of surgical treatment, the addition of preoperative therapies may be a novel add-on to surgery and postoperative CRT to control distant metastases. In addition, several clinical trials have reported that immune checkpoint inhibitors (ICIs) administered pre- or postoperatively improved pathological response and recurrence rates [19–21]. Several promising and prospective trials are currently investigating whether perioperative ICI therapy can improve survival, and promising results are expected [22]. Therefore, the introduction of ICI therapy in a perioperative setting may improve the prognosis of patients with postoperative high-risk factors through distant control.

This study had few limitations. First, this was a retrospective study conducted at a single institution with a small number of patients. Second, the concurrent postoperative CRT regimen, including fluorouracil, cisplatin, and S-1, was not uniform. However, the multidisciplinary team timely followed the guidelines in principle, and modified the treatment plan according to the patient’s general condition and socioeconomic status.

## Conclusion

The present study demonstrated that pLNs ≥ 3 and ENE were associated with poor prognosis of patients with LA-HPSCC who underwent primary surgical treatment. Besides primary surgical treatment followed by postoperative CRT, novel treatment modalities need to be developed for patients at high risks for distant metastasis, such as those with pLNs ≥ 3. Future research should investigate the use of ICI therapy preoperatively in combination with standard surgical treatment and postoperative CRT in patients with LA-HPSCC who have high-risk factors for recurrence.

## Data Availability

The data that support the findings of this study are available from Shizuoka Cancer Center, but restrictions apply to the availability of these data, which were used under license for the current study, and so are not publicly available. Data are however available from the authors upon reasonable request and with permission of Shizuoka Cancer Center.

## Declarations

Shizuoka Cancer Center Institutional Review Board approved this study (J2021-79-2021-1-3), and written informed consent was obtained from the patients.

## List of abbreviations

**CI**: Confidence interval.
**CRT**: Chemoradiotherapy.
**CT**: Computed tomography.
**DFS**: Disease-free survival.
**DMFS**: Distant metastasis-free survival.
**ECOG PS**: Eastern Cooperative Oncology Group performance status.
**EORTC**: European Organization for Research and Treatment of Cancer.
**ENE**: Extra-nodal extension.
**HNSCC**: Head and neck squamous cell carcinoma.
**HPC**: Hypopharyngeal carcinoma.
**HR**: Hazard ratio.
**ICI**: Immune checkpoint inhibitor.
**ICT**: Induction chemotherapy.
**LA-HPSCC**: Locally advanced hypopharyngeal squamous cell carcinoma.
**LN**: Lymph node.
**MRI**: Magnetic resonance imaging.
**ND**: Neck dissection.
**OS**: Overall survival.
**pLNs**: Pathological metastatic lymph nodes.
**RT**: Radiotherapy.
**RTOG**: Radiation Therapy Oncology Group.
**TPLE**: Total pharyngo-laryngo-esophagectomy.
